# High prevalence of low-allele-fraction somatic mutations in *STAT3* in peripheral blood CD8+ cells in multiple sclerosis patients and controls

**DOI:** 10.1371/journal.pone.0278245

**Published:** 2022-11-28

**Authors:** Miko Valori, Joonas Lehikoinen, Lilja Jansson, Jonna Clancy, Sofie A. Lundgren, Satu Mustjoki, Pentti Tienari

**Affiliations:** 1 Translational Immunology Research Program, University of Helsinki, Helsinki, Finland; 2 Neurocenter, Helsinki University Hospital, Helsinki, Finland; 3 Research and Development, Finnish Red Cross Blood Service, Helsinki, Finland; 4 Hematology Research Unit Helsinki, University of Helsinki and Helsinki University Hospital Comprehensive Cancer Center, Helsinki, Finland; CNR, ITALY

## Abstract

Somatic mutations have a central role in cancer, but there are also a few rare autoimmune diseases in which somatic mutations play a major role. We have recently shown that nonsynonymous somatic mutations with low allele fractions are preferentially detectable in CD8+ cells and that the *STAT3* gene is a promising target for screening. Here, we analyzed somatic mutations in the *STAT3* SH2 domain in peripheral blood CD8+ cells in a set of 94 multiple sclerosis (MS) patients and 99 matched controls. PCR amplicons targeting the exons 20 and 21 of *STAT3* were prepared and sequenced using the Illumina MiSeq instrument with 2x300bp reads. We designed a novel variant calling method, optimized for large number of samples, high sequencing depth (>25,000x) and small target genomic area. Overall, we discovered 64 *STAT3* somatic mutations in the 193 donors, of which 63 were non-synonymous and 77% have been previously reported in cancer or lymphoproliferative disease. The overall median variant allele fraction was 0.065% (range 0.007–1.2%), without significant difference between MS and controls (p = 0.82). There were 26 (28%) MS patients vs. 24 (24%) controls with mutations (p = 0.62). Two or more mutations were found in 9 MS patients vs. 2 controls (p = 0.03, p_corr_ = 0.12). Carriership of mutations associated with older age and lower neutrophil counts. These results demonstrate that *STAT3* SH2 domain is a hotspot for somatic mutations in CD8+ cells with a prevalence of 26% among the participants. There were no significant differences in the mutation prevalences between MS patients and controls. Further research is needed to elucidate the role of antigenic stimuli in the expansion of the mutant clones. Furthermore, the high discovered prevalence of *STAT3* somatic mutations makes it feasible to analyze these mutations directly in tissue-infiltrating CD8+ cells in autoimmune diseases.

## Introduction

Multiple sclerosis (MS) is an autoimmune disorder in which immune dysregulation in the periphery leads to central nervous system (CNS) myelin breakdown and axonal damage. MS is a multifactorial disease with both genetic and environmental risk factors.

Genetic approaches have identified a few hundred common polymorphisms that predispose to the development of MS [1, 2]. Collectively, these genetic association signals and their expression patterns suggest that peripheral leukocytes are the drivers of the perturbed immune response and that microglia may have a role in targeting the autoimmune process to the CNS [2]. Environmental contribution is important, and Epstein-Barr virus (EBV) has been recognized as the strongest risk factor of MS [3]. However, EBV infection is so common that the absolute risk associated with it is small, even after infectious mononucleosis [4]. EBV is an oncogenic virus that has also been implicated in lymphoid and epithelial cancers as well as lymphoproliferative conditions [5]. A recent analysis of the genetic and environmental risk factors of MS suggest that the known factors and their interactions cannot explain the low prevalence of MS (ca. 1/500 in high-risk areas) and disease pathogenesis is probably partly stochastic [6].

Somatic mutations resemble stochastic events, although the occurrence of mutations is not entirely random in codons [7]. Somatic mutations occur in human cells from embryonal stage over the entire lifespan and mutational burden increases with age [8]. Somatic mutations play a well-known major role in carcinogenesis but may also have a possible role in autoimmune disease by generating “a forbidden clone” that “can pass the homeostatic barrier although it is reactive with a self-component” as proposed already in the 1960s by Burnet [9]. Lymphoma driver mutations have been discovered in cells making pathogenic autoantibodies [10] and it has been proposed that somatic mutations in a self-reactive clone could bypass multiple tolerance checkpoints and lead to “stochastic onset of autoimmunity” [11]. Indeed, somatic mutations in peripheral blood leukocytes have been shown to underlie certain rare autoimmune disorders [12–14]. The role of somatic mutations in more common autoimmune diseases is, however, still unclear.

Somatic mutations in MS patients’ cultured autoreactive T-lymphocytes was reported in 1990s indirectly by using the hypoxanthine guanine phosphoribosyltransferase (HPRT) assay [15, 16]. We have recently shown that somatic mutations are preferentially detectable in CD8+ cells [17] and that these mutations are especially enriched in the signal transducer and activator of transcription 3 (*STAT3)* gene and to lesser extent in other genes implicated in hematological malignancies [18]. Activating mutations of *STAT3* can confer a survival and proliferation advantage to a CD8+ clone [19], increasing the clone size to reach detection threshold. Moreover, *STAT3* is a central component of several lymphocyte signaling pathways raising the question, whether the mutant CD8+ T cell clones play a role in autoimmunity.

*STAT3* is a cytosolic protein that, upon activation, is phosphorylated, dimerizes and translocates to the nucleus to activate or repress target gene transcription. *STAT3* can be activated by many pro-inflammatory cytokines, hormones and growth factors [20]. *STAT3* loss-of-function mutations have been reported in the human immunodeficiency condition termed Hyper immunoglobulin E syndrome; these patients are susceptible to bacterial and fungal infections as well as compromised T cell memory to varicella zoster virus and EBV [21]. Activating germline mutations of *STAT3* result in early-onset lymphoproliferation, immune deficiency and multi-organ autoimmunity [22, 23]. Somatic mutations activating *STAT3* have been discovered especially in T-cell large granular lymphocytic (T-LGL) leukemias (40%) as well as in Felty syndrome (neutropenia, splenomegaly, rheumatoid arthritis) and less frequently in aplastic anemia and myelodysplastic syndrome [24–27]. Interestingly, in aplastic anemia the presence of *STAT3* mutations was associated with the carriership of HLA-DR15, which is a major genetic risk factor of MS [26]. Activating *STAT3* mutations cluster in the Src-homology-2 (SH2) domain and lead to constitutive phosphorylation, dimerization and upregulation of transcriptional activity [24]. T-LGL leukemia patients with *STAT3* mutations often presented with rheumatoid arthritis [28] providing a link between autoimmunity and somatic mutations in *STAT3*.

*STAT3* has been implicated in many ways in MS. Germline intronic variants and a risk haplotype encompassing 5’ half of the *STAT3* gene and its immediate promoter region have been found to predispose to MS [29]. The *STAT3* gene as well as the JAK-STAT signaling pathway have been identified important in protein interactome analyses of published GWASes [30, 31]. Moreover, increased *STAT3* phosphorylation has been associated with disease activity of MS [32, 33]. *STAT3* signaling has been implicated in the skewed effector:regulatory T cell balance in both MS and experimental autoimmune encephalomyelitis (EAE), the most widely used animal model of immune-mediated demyelination. In the context of EAE, there is evidence that inhibition of *STAT3* directly [34], or upstream by targeting the JAK1/2 pathway [35] can ameliorate disease symptoms.

In the present study, we analyzed peripheral blood CD8+ cells’ DNA of MS patients and matched controls by deep amplicon sequencing and searched for somatic mutations in the two main exons encoding the SH2 domain of *STAT3*. In previous research by us and others, the SH2 domain has contained activating somatic mutations in various conditions and is thus a promising target for screening. By limiting our sequencing to these two exons that especially our previous study [18] flagged as promising, we were able to employ very high sequencing depths. The high sequencing depth allowed us to discover smaller clones that are far more numerous than large, clearly expanded cell clones (allele fraction >1%) detectable using less sequencing depth [36], opening a view to the landscape of *STAT3* somatic mutations previously unseen. We aimed to estimate the prevalence of *STAT3* somatic mutations and to test, if the *STAT3* mutations are distributed differently between MS cases and controls. It is known that high-depth variant calling may prove challenging [37], so to better facilitate the high sequencing depths in this data, we developed a novel variant calling method that is optimized for large number of samples, high sequencing depth and small target genomic area.

## Materials and methods

### Study participants

MS patients were recruited at the Helsinki University Hospital Department of Neurology outpatient clinic during their diagnostic examinations (n = 66), or during their follow-up after MS diagnosis (n = 32). We included patients with longer duration to increase the age range of the subjects. All patients fulfilled either the diagnostic criteria of Poser (n = 11), McDonald 2001 (n = 84), or from 2018 onwards McDonald 2017 (n = 3). Cerebrospinal fluid oligoclonal bands or increased IgG index were found in 97/98 (99%) of the patients. Barkhof’s [38] magnetic resonance imaging 4/4 criteria were met in 65 (66%) of the patients in their diagnostic scan, ¾ criteria in 19 (19%), 2/4 criteria in 11 (11%) and ¼ criteria in 3 (3%). Age- and sex-matched controls (n = 99) without evidence for demyelinative disease were selected from patients with other neurological diseases/symptoms visiting the outpatient clinic (n = 19), blood donors (n = 38) and healthy volunteers (n = 42). The demographic features of the patient and control groups are shown in Table 1 and individual information of the MS patients in S1 Table and controls in S2 Table. S2 Table also shows diagnoses/ICD-10 codes, CSF and/or MRI examination results (normal vs. specific finding) of the 19 controls recruited from the patients visiting the outpatient clinic. The diagnoses of these 19 controls were paresthesia (n = 6), previous neurosarcoidosis (n = 2), intervertebral disc disease (n = 2) and one case of each of the following: spinocerebellar ataxia, metabolic encephalopathy, myasthenia gravis, unspecific myasthenia plus Meniere’s disease, narcolepsy, pseudotumor cerebri, limb pain, subjective visual disturbances, unspecific vertigo. Differential diagnostic workup included CSF and/or MRI examinations and none had evidence of CNS inflammation in these examinations. Of the 98 patients 94 (96%) were ethnic Finns; additionally, there were two patients that were British, one Estonian, and one Polish. Of the 99 controls 97 (98%) were ethnic Finns, additionally, one control was born in South Korea and one in Poland. Four of the 98 MS patients were excluded from the mutation analysis, because their samples had low sequencing coverages. The number of patients in the tables is therefore 94. Some of the participants were included in our previous studies as well using much lower sequencing depths [17, 18]. Of the MS patients 16 were included in our first study [17] and 17 in our second study [18]. Of the controls 2 were included in our first study [17] and 20 in our second study [18]. In the first study using a median coverage of 723x we disocovered one *STAT3* SH2 domain mutation in 20 participants and in the second study using a median coverage of 2349x we discovered 6 *STAT3* mutations in 42 participants, of which 4 were in the SH2 domain.

**Table 1:** Demographic features of the participants included to the mutation analysis.

	MS patients	Controls
**Number**	94**	99
**RMS/SPMS/PPMS**	80/10/4	-
**Median age (range)**	37 yrs (20–68)	35 yrs (19–70)
**Mean age**	39.4 yrs	39.8 yrs
**Percentage females**	72%	71%
EDSS* mean/median (range)	2.4/2.0 (0–6.5)	-

RMS, relapsing MS; SPMS, secondary progressive MS; PPMS, primarily progressive MS;

*EDSS, Expanded disability scale score at the time of sampling.

**Four MS patients were excluded from the mutation analysis, because their samples had low sequencing coverages.

This study has been approved by the regional ethics committee (Dno 83/13/03/01/2013). All participants gave informed written consent.

### CD8+ cell separation and DNA extraction

Peripheral blood mononuclear cells (PBMCs) were extracted from venous EDTA blood using Ficoll-Paque PLUS (GE Healthcare) as previously described [18]. From the PBMCs, positive separation with MACS CD8 antibody MicroBeads (Cat. No. 130-045-201, Miltenyi Biotec, Bergisch Gladbach, Germany) was performed using an OctoMACS magnetic separator (Miltenyi Biotec) following the manufacturers protocol. From the separated cell populations, DNA and RNA were extracted using the InviTrap Spin Universal RNA Mini Kit for the amplification and library preparation in batches 1–3 (Stratec Biomedical, Birkenfeld, Germany) according to manufacturer’s instructions. From the blood donor samples, DNA was extracted with Nucleospin Tissue DNA extraction kit (Machery Nagel, cat. no 740952.250) according to the manufacturer’s instructions. To obtain better yield of DNA the QIAamp DNA Mini Kit (Qiagen) was used for batch 4. The purities of the separated CD8+ cells were tested in 52% of the 197 samples by flow cytometric analysis, in which T cells (CD3+) represented 89–99% of the cells and the observed purities for CD8 were all ≥ 84%. The CD8 subpopulations (CD27 and CD45RA positives and negatives) were not tested for purities, because of the small number of cells left after sequential negative and positive selection with immunomagnetic beads.

### HLA-DR15 typing

We used three methods. In 67 participants (34 MS patients, 33 controls) HLA-DR15 was genotyped by imputation from the Illumina Global Screening Array-24+MD v2 BeadChip data as previously described [39]. In the 36 of the 38 blood donor controls HLA-DR15 was typed at the Histocompatibility Testing Laboratory, Finnish Red Cross Blood Service accredited by European Federation for Immunogenetics, as previously described [40]. In 87 participant (56 MS patients, 28 controls) an allele-specific PCR method was used [41]. HLA-DR15 data was missing in 6 participants (4 MS, 2 controls). HLA-DR15 was found in 50% (45 out of 90) of the MS patients and in 30% (29 out of 97) of the controls.

### *STAT3* SH2 domain exon 20 and 21 amplicon sequencing and library preparation

We prepared amplicons using 500ng of template DNA targeting the exons 20 and 21 of STAT3 in the same PCR reaction. Duplicate PCR reactions were made in all controls and in 82 of the 98 MS patients. CD8+ cell DNA was used up in 16 cases and only single amplicon was made from these (all four excluded patients had sequence from only one PCR reaction hence the final mutation analysis included 12 MS patients with single amplicon data). For exon 20, we used the target specific forward primer sequence 5’-GTCAAGGCCATCTCCACCC and the reverse primer sequence 5’-GGGATGGATGCCCTGTTAGC. For exon 21 the forward primer sequence was 5-‘AATGCCAGGAACATGGAAAATC and the reverse primer was 5’-TCTTTCCTTCCCA TGTCCTGTG. We followed the Illumina 16S sequencing protocol, and the primers contained 5’ overhang sequences used for sequencing adapter attachment, followed by the above target specific sequences. Amplicons from different samples and replicates were distinguished by separate index sequences, but the two different exons were pooled together for PCR amplification and shared a common index sequence. After library preparation, the amplicons were sequenced using the Illumina MiSeq instrument producing 2x300bp reads.

In addition to each participant’s CD8+ cell DNA, we also prepared amplicons from two technical reference sources: DNA from the NA12878 cell line (Coriell Cell Repository, Camden, NJ, USA), and from a DNA pool mixed from whole blood Helsinki biobank samples of 8 healthy subjects age <35 yrs without autoimmune disease or cancer (https://www.helsinginbiopankki.fi/en/front-page).

The amplicon sequencing was split into four different MiSeq sequencing batches. Total number of sequenced libraries was 449, 78 libraries were sequenced in two or more batches to increase sequencing depth.

### Data analysis

Sequenced reads from the PCR amplicons were mapped to the GRCh37 reference genome using BWA MEM [42]. Quality control was performed by assessing read quality scores with the aid of seqtk and sequencing depths using mosdepth [43]. Germline variants were called using GATK HaplotypeCaller [44].

Detecting low allele fraction somatic mutations from standard next generation sequencing data is known to be challenging using current methods [37, 45]. As several of our sequencing libraries reached depths well over 25000x, we had an opportunity to search for very low allele fraction variants, but as current methods have limitations [37] we developed a new variant calling software to enable robust somatic variant calling from our high depth amplicon data. The full source code of the variant caller is available for inspection and free use at https://github.com/mvalorilab/somaticbatch under an open source license. The key difference of our variant caller from existing methods is that it is meant to analyze a full library preparation and sequencing batch at a time. It can then attempt to remove repeating low frequency artefacts that are specific to a given batch, which would not be possible if calling variants from a single sample, without knowledge of the batch’s error profile. In addition to noise generated during sequencing, library preparation artefacts represent a major source of errors in Illumina next generation sequencing data [46], and to help differentiate these sources of error from true biological variation, awareness of other samples data in the same batch enables checks for repeating artefacts that can be batch-specific. The robustness of our variant calling method is examined in the discussion section, based on our variant calling results.

For each run of the variant caller, sequencing data from libraries of a single library preparation and sequencing batch were analyzed together. For each sample within a batch, only such basecalls that were identical on both strands of the sequenced amplicon and had a combined base quality score of at least 60 were accepted. Each amplicon was paired-end sequenced using sufficiently long reads, so data to check base calls on both strands from the corresponding paired end reads was available. Within a single variant calling run, two statistical tests were performed at each coordinate, for each sequencing library, and for every possible base substitution. Both of these tests and a third criteria for sequencing noise all had to be passed for any somatic variant call to be included in our results, as detailed below.

To eliminate repeating artefacts withing a batch, a between-sample comparison using Fisher’s exact test was used to compare the number of reference and alternate allele bases of a sequencing library to the corresponding summed base counts of all other sequencing libraries at that genomic coordinate. This criterion demands that a somatic variant call must be present in enough reads to distinguish it from the locus specific background noise represented by the summed base counts obtained from all other libraries.

Then, a separate within-sample test was performed with the aim to eliminate spurious false positive calls from more poorly performing libraries that present an overabundance of some particular base substitution type. The rate of similar base substitutions in other coordinates of the sample was calculated, and a binomial test was performed using this expected noise rate and the observed number of alternative bases at this particular coordinate.

The p-values obtained from both tests were Bonferroni corrected by the total number of putative somatic variant calls. For each variant call, a third statistic to distinguish true variation from noise was calculated as the log10 ratio of the fraction of variant bases observed to the background noise rate (calculated identically as for the within-sample test). We refer to this statistic as log noise ratio.

**Fig 1:**
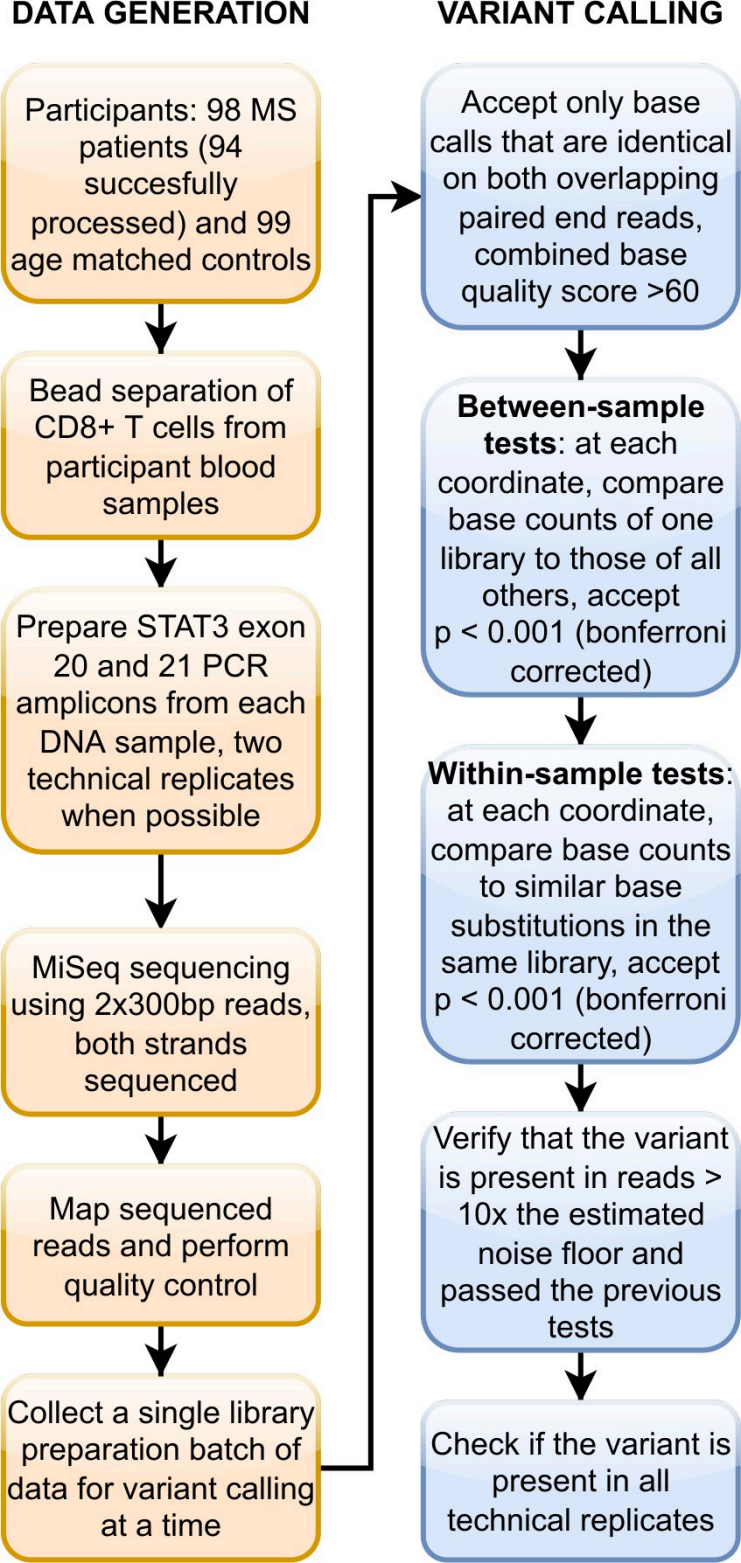
Study workflow.

In order to obtain high sensitivity and specificity, we set the cutoffs for our p-values by demanding that none of our technical replicate libraries (n = 28) showed any variant calls using the same parameters as all other samples. We arrived to the parameters of between-sample p < 0.001 (after bonferroni correction), within-sample p < 0.001 (after bonferroni correction) and log noise ratio > 1. The variant calling procedure is summarized in Fig 1. Observations that support the specificity of our somatic variant calling procedure are detailed in the discussion section.

### Double-positive and single-positive criteria in variant calling

For the most stringent variant call set, we required that a variant call needed to be present in two separate libraries from the same donor DNA (double-positive). We made analyses also with less stringent criteria by allowing a statistically significant call be present in only one library (single-positive). This was carried out to maximize the utilization of the data since we did not have duplicate libraries from 16 MS patients and all libraries did not produce equally good coverage.

### Statistical analysis of demographic and laboratory data in mutation carriers vs. non-carriers

Univariate analyses were carried out using student’s t-test or Fischer’s exact test. In the multivariate analysis, using binary logistic regression, we calculated the relationships between variables that showed nominal statistical significance (p<0.05) in the univariate analysis. Neutrophil count was analyzed in the multivariate analysis, leukocyte count was omitted, because it is highly dependent on neutrophil count. BMI and smoking were included to the multivariate analysis due to their possible effect on neutrophil count. SPSS Statistics, Version 25v100 (IBM, USA) was used in these analyses.

## Results

### *STAT3* amplicon sequencing coverages and failure rates

We have previously shown that blood leukocyte clones with somatic mutations are detectable especially in the CD8+ cells [17] and mutations show enrichment in the *STAT3* gene [18], suggesting more detailed screening at this locus. Here we used deep amplicon sequencing to call low allelic fraction *STAT3* somatic mutations from the CD8+ cells’ DNA in a larger set of MS cases and controls with much improved sequencing depth compared to our previous studies. The overall workflow is shown in Fig 1.

We recruited MS patients (n = 98) at the Helsinki University Hospital Department of Neurology outpatient client, and age- and sex matched controls (n = 99) composed of other neurological disease patients from the same clinic, blood donors, and healthy volunteers. Details of the participants are described in the methods section. From peripheral blood we separated CD8+ cells and prepared targeted amplicons for next generation sequencing.

The two main exons (20 and 21) encoding the *STAT3* SH2 domain were targeted. Counting all technical replicates, 449 libraries were sequenced in 527 sequencing reactions (215 sequencing reactions in MS patients, 284 in controls, and 28 in reference DNAs). A median sequencing depth of 74,552x (range 0–379,019x) was obtained for exon 20 amplicons, and a median depth of 46,912x (range 0–365,883x) for exon 21 amplicons. In exon 20, ≥25,000x coverage was reached at least once in 94 of the 98 MS patients and in all 99 controls samples., In exon 21, ≥25,000x coverage was reached at least once in 91 of the 98 MS patients and 93 of the 99 controls. The coverages in the most successful sequencing reactions in patients and controls are illustrated in S1 Fig.

The coverages in all sequencing reactions (including failures) in patients and controls are illustrated in S2 Fig. In four participants repeated failures were encountered, all were MS patients. These MS patients had coverages <1000x in all sequencing runs and we eventually ran out of CD8+ cell DNA. These cases were excluded leaving 94 MS patients and 99 controls for the mutation analyses. The coverages, number of libraries and sequencing batches/runs of each participant are listed in S3 Table. The failures were related to PCR amplification in library preparation that takes place before actual sequencing, as less successful libraries could already be observed during library quantification.

### Somatic variant calling using double- and single-positive criteria–overall data

We called somatic variants from the amplicon sequencing data using a custom variant caller optimized for data of this type. Because we prepared two technical replicate amplicons from the majority of our DNA samples, we were able to verify several variant calls using two independent sequencing libraries from the same donor (double-positive criteria). However, we did not have enough DNA for two amplicons in all MS patients included to the mutation analysis and all libraries did not obtain high enough sequencing coverage for robust variant calling. Therefore, we also used less stringent criteria, which require that statistically significant variant call is present in one library (single-positive criteria). This analysis allowed us to include more samples with high sequencing depth, with a potential cost of increased noise.

The results using both criteria are summarized in Table 2. No somatic variants were present in technical reference DNAs (see Methods; *STAT3* SH2 domain exon 20 and 21 amplicon sequencing and library preparation) using the same quality cutoffs that we used for the participant DNA amplicons. The coverages, counts of reference/variant alleles, sequencing batch/run number, predicted amino acid changes, codon context and statistics of all discovered variants are listed in S4 Table. The median coverage at the sites of the discovered variants was 47,288x and the median number of reads of the discovered somatic variants in the sequencing runs (alt allele) was 28.

**Table 2:** Overall mutation characteristics using double-positive and single-positive criteria (all participants included).

	Double-positive	Single-positive	Total
Number of subjects	22	28	50
Number of variants	26	38	64
Median allele fraction	0.13%	0.037%	0.065%
Allele fraction range	0.034%-1.2%	0.007%-0.90%	0.007–1.2%
Non-synonymous,	26 (100%)	37 (97%)	63 (98%)
Synonymous	0	1	1
Found in COSMIC/OMIM*	96%	63%	77%
Carrier vs. non-carrier age**	48.5 vs. 38.7 yrs	45.4 vs. 37.3 yrs	47.1 vs. 37.3 yrs
p-value for age effect	0.0031	0.0032	2.36 x 10E-5

*Somatic mutation described in cancer or germline mutation implicated in autoimmunity. COSMIC, Catalogue Of Somatic Mutations In Cancer; OMIM, Online Mendelian Inheritance in Man.

**Median age, carrier vs. non-carrier refers to participants with or without a detected mutation. Twelve individuals (MS patients) had only one sequencing library available and couldn’t have reached double-positivity, 2 of them had a single-positive finding.

Using both criteria the mutation type was extremely non-random as 63 of the mutations were non-synonymous, while only one was synonymous. Using both criteria, there was a significant difference in the mean age of participants with vs. without a *STAT3* mutation (Table 2), in line with what has been shown between somatic mutation load and age in general [47]. These results suggest that the mutations are very likely true rather than sequencing noise.

Overall a total of 64 somatic variants were detected and these were present in 50 (26%) of the 193 participants. There was no statistical difference between the female and male participant groups’ mutation carrier status (p = 0.36, Fisher’s exact test).

We analyzed correlation between the allele fractions of the discovered variants and age. A non-significant trend (r = 0.19; p = 0.088) towards higher allele fraction with increasing age was observed (S3 Fig). There was no significant difference in the number of mutations between different subsets of participants in the control group after controlling for age (linear regression, volunteer subset p = 0.96, blood donor subset p = 0.55).

No germline variants/polymorphisms were detected within *STAT3* exons 20 and 21 in any of the participants.

### *STAT3* somatic mutations in MS patients and controls

All mutations found in the 94 MS patients and 99 controls are shown in Table 3. The detailed breakdown of mutations based on their detection using either double- or single-positive criteria is shown in S5 Table. The number of different mutations was 23 in MS patients and 12 in controls. The mutation load was non-significantly higher in MS group than in controls (38 vs. 26, Mann-Whitney U test p = 0.35). The median allele fraction was 0.066% (range 0.007–1.2%) in patients and 0.056% (range 0.014–1.2%) in controls (p = 0.82 for difference, student’s t-test).

**Table 3:** Discovered *STAT3* somatic mutations in 94 multiple sclerosis (MS) patients and 99 controls (Ctrl).

Mutation	Exon	COSMIC/OMIM	CADD score	MS (n)	Ctrl (n)
S590R	20	-	26,1	**1**	**0**
K591X	20	-	33	**1**	**0**
E592*	20	-	44	**1**	**1**
K601T	20	-	24,8	**1**	**0**
T605X	20	-	31	**2**	**0**
F606X	20	-	33	**1**	**0**
S614R	20	COSV52888203	29,4	**9**	**5**
E616*	20	-	43	**0**	**1**
E616del	20	COSV52882850	22,9	**1**	**0**
G617G	20	-	12	**1**	**0**
G618R	20	COSV52882950	32	**1**	**4**
P639Q	21	-	26,4	**1**	**0**
Y640F	21	COSV52882807	23,8	**3**	**1**
Q644H	21	-	23,7	**1**	**0**
N646K	21	OMIM #615952	23,3	**1**	**0**
N647I	21	COSV52882818	23,7	**0**	**1**
Y657dup	21	COSV52891910	21,9	**1**	**1**
K658N	21	COSV52886038 OMIM #615952	26,4	**1**	**2**
K658M	21	COSM1166797	28,8	**0**	**1**
K658R	21	COSV52886492	25	**1**	**1**
I659L	21	COSV52891525	24,4	**2**	**0**
D661Y	21	COSV52882933	25,3	**4**	**5**
D661V	21	COSV52886283	24,4	**1**	**3**
L666V	21	-	25,3	**1**	**0**
V671L	21	-	24	**1**	**0**
E690*	21	-	38	**1**	**0**
Total				**38**	**26**

*denotes stop mutation; COSMIC, Catalogue Of Somatic Mutations In Cancer; OMIM, Online Mendelian Inheritance in Man; CADD, Combined Annotation Dependent Depletion. Based on CADD’s recommendation a CADD score >20 can be considered predictably deleterious (https://cadd.gs.washington.edu/info). Based on Ensembl’s recommendation CADD scores >30 are likely deleterious. Variants with scores >30 are predicted to be the 0.1% most deleterious possible substitutions in the human genome (https://www.ensembl.org/info/genome/variation/prediction/protein_function.html).

There were 26 MS patients and 24 controls with mutations (Table 4), and no statistically significant difference in the *STAT3* mutation prevalence was observed between the cohorts (p = 0.62, Fisher’s exact test). There were 9 subjects with two or more mutations in the MS group vs. 2 in the controls (p = 0.03, p_corr_ = 0.12, Fisher’s exact test).

**Table 4:** Number of *STAT3* mutation carriers in MS patients and controls.

	MS (n = 94)	Ctrl (n = 99)	p-value*
Any mutation	26 (28%)	24(24%)	0.62
Median age	49 yrs	47 yrs	
Any mutations ≥2	9 (9.6%)	2 (2.0%)	0.03 (p_corr_ = 0.12)
Median age	50 yrs	58 yrs	
Mutation in COSMIC/OMIM	19 (20%)	22 (22%)	0.86
Median age	51 yrs	51 yrs	
Other mutations**	9 (9.6%)	3 (3.0%)	0.08 (p_corr_ = 0.24)
Median age	48 yrs	39 yrs	

* Fisher’s exact test, corrected, when necessary by the number of comparisons (4);

**Mutation not found in COSMIC/OMIM. COSMIC, Catalogue Of Somatic Mutations In Cancer; OMIM, Online Mendelian Inheritance in Man.

### Mutations in CD8+ cell subtypes

We had DNA samples at different time points and from CD8+ subfractions from four of the participants (3 MS cases and 1 control), who had somatic mutations in the *STAT3* SH2 domain in our previous studies [17, 18]. These mutations were initially discovered at allele fraction ≥ 0.4%. Here we analyzed *STAT3* mutations in the following CD8+ cell subtypes: central memory (CM, CD27+), effector memory (EM, CD27-, CD45RA+) and terminally-differentiated effector memory (TEMRA, CD27-, CD45RA-). We wanted to address the questions whether the mutations are enriched in a specific subfraction of CD8+ cells and are the clones persistently detected.

**Fig 2:**
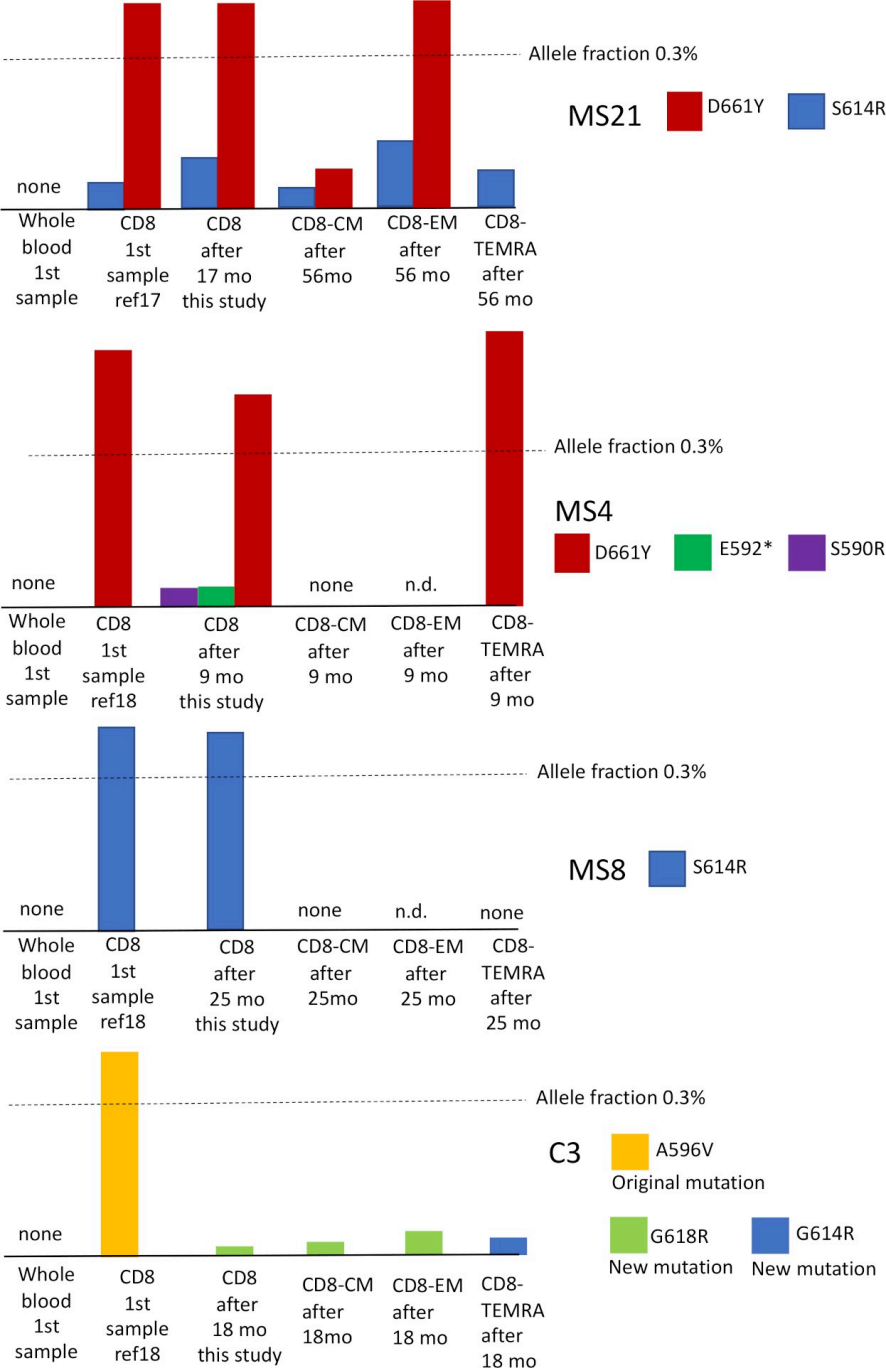
Follow-up analysis of somatic mutations in the *STAT3* SH2 domain in total CD8+ cells and CD8+ subtypes. CD8 = total CD8+ cells, CM = central memory (CD27+), EM = effector memory (CD27-, CD45RA+), TEMRA = terminally-differentiated effector memory (CD27-, CD45RA-), mo = months.

The results are summarized in Fig 2. Based on this small dataset, it can be seen that the CD8 subfractions with mutations were variable. MS-21 had two detectable mutations, one (S614R) was found in all subfractions, while the other (D661Y) was found in all, except TEMRA. These mutations are likely present in different clones, because their allele fractions and distribution in subfractions were different. MS4 had initially only one mutation detectable by the gene panel sequencing (D661Y); by amplicon sequencing we discovered two additional mutations (E592* and S590R) with very small allelic fractions. D661Y was present in TEMRA and was not found in CM (EM was not tested). The two additional mutations were clearly independent from D661Y. MS8 had one mutation (S614R), which was not detectable in CM or TEMRA (EM was not tested). C3 had initially one mutation (A596V), which was not detectable after 18 months. However, another mutation was discovered (G618R), which was present in CM and EM compartments but not in TEMRA. A third mutation was discovered selectively in TEMRA (G614R). Overall CM fraction had either no detectable mutations or smallest allele fractions. Two of the mutations were not found in TEMRA, while one of the mutations was found only in TEMRA (in C3).

All mutations found in the first sample were discovered also in follow-up in MS patients. The A596V mutation found in the first sample in control C3 was not detectable in follow-up; however, two additional mutations had emerged.

It is of note that none of the mutations were detectable in whole blood DNA. This is consistent with the view that the mutations belong exclusively to CD8+ clones, and the mutant clones represent such a small fraction of whole blood cells that they fall under the detection limit.

### Comparison of *STAT3* mutation carriers and non-carriers among MS patients

Mutation carriers were found in 25% of the relapsing MS (RMS) patients, 50% of secondary progressive MS (SPMS) patients and 25% of primary progressive MS (PPMS) patients. All patients were pooled for this analysis [SPMS (n = 10) and PPMS (n = 4) groups were too small for statistical comparisons].

We compared characteristics of MS patients with or without mutations in univariate and multivariate analyses (Table 5). In univariate analysis, age (p = 0.0001), disease duration (p = 0.01), use of medication (0.01), total leukocytes (p = 0.009) and neutrophils (p = 0.006) associated with mutation carrier status.

**Table 5:** Phenotypic comparison of *STAT3* mutation carriers vs. non-carriers among MS patients.

	Any mutation (n = 26)	No mutation (n = 68)	Univariate p-value	Multivariate p-value	missing data (n)
Females (n)	18 (69%)	50 (74%)	0.80	0.165	-
Age at exam (median)	49 yrs	36 yrs	**0.0001**	0.019	-
Duration from Dg (months, mean, range)	91 (0–450)	34 (0–288)	**0.011**	0.75	-
EDSS (mean)	2.5	2.2	0.45	-	-
Use of DMT (n)	10 (38%)	9 (13%)	**0.010**	0.56	-
BMI (mean, range)	25.8 (20–38)	25.7 (17–37)	0.89	0.80	14
Current smoker (n)	7/23 (30%)	12/65 (18%)	0.25	0.031*	6
Never-smoker	12/23 (52%)	37/65 (57%)	0.80	-	6
MRI (Barkhof at Dg) (mean)	3.6	3.4	0.43	-	-
HLA-DR15 +	12/25 (48%)	33/65 (51%)	1.0	-	4
CSF parameters					
OCB positive (n)	26 (100%)	67 (99%)	1.00	-	-
IgG index (mean)	0.98	1.08	0.47	-	12
Leukocytes (mean)	13	9.54	0.37	-	7
Protein (mean)	413	360	0.11	-	7
Blood parameters (mean)					
Hemoglobin	138	140	0.65	-	2
Erythrocytes	4.61	4.62	0.92	-	2
Trombocytes	253	283	0.055	-	2
Total leukocytes	6.2	7.4	**0.009**	-	2
Neutrophiles	3.07	4.09	**0.006**	0.019	7
Lymphocytes	2.15	2.19	0.82	-	7
Monocytes	0.58	0.61	0.45	-	7
ALAT	24	27	0.49	-	3
ESR	7	6	0.67	-	24
CRP (abnormal, n)	5 (19%)	16 (24%)	1.0	-	4

Fisher’s exact test was used to calculate p-values for categorial variables, students t-test was used for quantitative variables. Dg = diagnosis, EDSS = expanded disability status scale, DMT = disease modifying treatment (any pharmaceutical therapy used to treat MS), BMI = body mass index, ALAT = alanine aminotransferase, ESR = erythrocyte sedimentation rate, CRP = C-reactive protein. Multivariate analysis was performed by logistic regression; regression analysis included the following variables; sex, age, duration, use of DMT, BMI, current smoking and neutrophils. BMI and smoking were included, because of their possible effect on neutrophil counts.

*In multivariate analysis smoking was dichotomized to current smokers and never-smokers.

Multivariate analysis using logistic regression indicated that many of the differences in univariate analysis between mutation carriers vs. non-carriers were secondary to age (multivariate p = 0.019). However, lower neutrophil count was still associated (p = 0.019) with mutation carrier status (Table 5). Neutropenia has been previously reported in carriers of *STAT3* gain-of-function mutations [25]. Current smoking was associated with mutation carrier status only in the multivariate analysis (p = 0.031). In this analysis smoking was dichotomized to current smokers and never-smokers. The details of the logistic regression analysis are shown in S6 Table. Contrary to the observation in aplastic anemia [27], we did not find any association of *STAT3* somatic mutation carriership with HLA-DR15.

## Discussion

In this work, we detected somatic *STAT3* SH2 domain mutations in peripheral blood CD8+ T cells in 26% of the study participants. This mutation positivity rate may appear high, because our participants did not have any obvious disorder associated with somatic *STAT3* mutations. A clear difference between the mutations discovered in this study and those reported in previous studies is that the mutations in our study were detected at much smaller allele fractions (range 0.007–1.2%). In previous studies, *STAT3* somatic mutation allele fractions in T-LGL leukemia have been typically >15% [28], in Felty syndrome 1–9% [25], in myelodysplastic syndrome 0.6–13%, and in aplastic anemia on average 4.5% [27]. Only in T-LGL leukemia and Felty syndrome the reported mutation prevalences been higher than the 26% observed in our study [28]. Most of the somatic mutations detected in our cohort (77%) were known variants implicated in hematological cancers or lymphoproliferative disease. Therefore, it is important to consider the robustness of our detection process and discuss first whether the findings are indeed real.

Detecting low allele fraction somatic mutations in typical next-generation sequencing data using currently available variant calling methods is known to be challenging. Starting from an allele fraction below 10% problems in performance begin to appear, and when approaching 1%, most of the true positive variants may be missed by the widely used “gold-standard” variant callers when specificity is kept at an acceptable level [37, 45]. Causes of challenges for low allelic fraction variant calling include errors in sequencing, and most of all artefacts that arise during library preparation steps, such as PCR [46, 48]. Distinguishing these low frequency artefacts from true low frequency biological events is a major challenge. Solutions to reliably reach lower allelic fractions include different specialized molecular barcoding techniques [8, 49] and single cell sequencing [50]. However, these options may be prohibitive in cost and many of them require difficult laboratory protocols. Our samples were sequenced using standard low-cost methods but to very high depths (≥25000x), and thus necessitated a special method for data analysis, which we developed. The key difference of our method in comparison to existing ones is that it analyses a single library preparation and sequencing batch of samples at a time and can thus attempt to eliminate systematic low frequency library preparation and sequencing artefacts specific to that batch, as they tend to repeat between samples.

We are confident that the somatic mutations detected by our method are real ones and not due to errors in detection. Multiple arguments support this conclusion as the somatic mutations that we detected showed a distribution consistent with real biological events instead of technical noise in several ways. First, the mutations were overwhelmingly non-synonymous (98%) and the majority (77%) were previously observed variants in COSMIC/OMIM, with only one mutation being synonymous. This suggests a functional role and a possible growth advantage conferred by the mutations, whereas random technical noise would be more evenly distributed between synonymous and non-synonymous variant types. Second, the mutation carriership associated strongly with age, a phenomenon one would not expect, if the variants were noise. Third, we detected no somatic mutations in the libraries derived from technical control DNA (n = 28). Instead, the mutations were only present in the separated CD8+ cell samples. Possible sequencing noise (or contamination) would be expected to be present in the technical control libraries as well, because these were processed along the participant libraries. Fourth, in the cases where we were able to sequence two technical replicate libraries to a high sequencing depth, the detected somatic mutations were typically present in both replicates (double-positive). When variant call was present in one library only (single-positive) the mutation type was also non-random (97% non-synonymous) and mutation carriership associated with age. Fifth, neutrophils were slightly, but significantly, lower in the mutation carriers consistent with a real biological effect and in line with reports in T-LGL leukemia and Felty syndrome [24, 25].

Because our cohort included both MS patients and controls and the somatic mutations were present in similar frequencies in patients and controls, we conclude that CD8+ cell *STAT3* somatic mutations with low allele fraction are common in the CD8+ cells in the general population. The true rate can even be higher than our 26%, a more fine-grained cell separation or a more advanced sequencing method could detect further mutations at smaller allele fractions (as shown in Fig 2 in donor C3, who had two new mutations, one of which was detectable only in CD8+ TEMRA fraction). We cannot present an exact detection limit for our method because it varies by the noise level posed by different sites and substitution types in each sequencing run, but the lowest allele fraction reported in this study for a single nucleotide variant was 0.007%.

What is the biological or clinical significance of the mutations? Somatic mutations in the SH2 domain of *STAT3*, such as S614R, D661V and D661Y, are known to be activating and prevalent in patients with solid and hematological malignancy. These mutations were detected in multiple participants in this study, both in MS patients and controls. Our participants, however, did not have cancer or a hematological malignancy. It also seems unlikely that a large part of our mutation positive participants would develop such a malignancy later, given the low incidence of *STAT3* mutation related conditions in comparison to the high positivity rate of mutations in this study. This suggest that the presence of *STAT3* mutation as such does not necessarily lead to large T cell expansion, but for example a second hit or antigen stimulus is required. Human T-cell leukemia virus type-2 (HTLV-2) infects predominantly CD8+ cells and may play a role in the development of T-LGL leukemia in some cases [51]. Recently it was found that 4 out of 30 (13%) asymptomatic HTLV-2 infected subjects had *STAT3* SH2 domain mutation with allele fraction 0.5–12% [52]. Possibly with more sensitive method and lower allele fraction cutoff, more mutations could have been detected in HTLV-2 infected subjects. In comparison, in our data only 6 participants (3%) had variant allele fraction ≥0.5%. It is possible that chronic viral infections or other antigen stimuli exert selection pressure on the mutant clones, in addition to the mutation itself, resulting in their expansion and detection.

How do these findings relate to clonal hematopoiesis (CH)? CH is an age-related phenomenon in which mature blood cells are derived from single or a few dominant hematopoietic stem cell lineages, which typically have somatic mutations that confer selection advantage [53]. CH can be divided into lymphoid and myeloid arms with different driver mutations; *STAT3* is not among the typical drivers in lymphoid CH [54]. In CH the mutant clones usually reach variant allele fraction >2% in whole blood and can be found in more than 10% of >70-year old individuals. By using more sensitive methods it has been found that 19/20 (95%) of healthy donors aged 50–75 yrs had mutations in the typical myeloid driver genes at a median variant allele fraction of 0.24% in the buffy coat DNA [55]. CH is associated with increased risk of hematological cancer, cardiovascular disease and infections [53]. The mutations we found probably do not represent CH. It seems likely that the majority of the *STAT3* mutations do not evolve from the hematopoietic stem cell level but later in T cell development. The low variant allele fractions in general, lack of mutation detection in whole blood and the different distributions of the mutations in the CD8 subtypes among the donors with higher variant allele fractions (Fig 2) favor this interpretation. More data is nevertheless needed to prove this, using for example paired bone marrow and blood samples.

As to the biological effect of the mutations, we detected a modest but statistically significant decrease in neutrophils in the MS patients with mutations vs. non-carriers, consistent with reports of neutropenia in *STAT3* mutation related T-LGL leukemia and Felty syndrome [24, 25]. Although the *STAT3* somatic mutation carrier frequency was similar between MS patients and unaffected controls, we found a modest (p = 0.03, p_corr_ = 0.12) increase in the number of participants with multiple mutations. This finding should be regarded as nonsignificant, because interindividual differences in the sequencing depth affect the chances of finding multiple mutations. As somatic *STAT3* mutation prevalences were not significally different between MS cases and controls, it is clear that just the presence of a detectable mutation in an arbitrary CD8+ T cell clone is not a risk factor for MS. A limitation of this study is that we did not assess more precisely the phenotypes of the sequenced CD8+ cells. It is also of note that MS typically starts in early adulthood, but more somatic *STAT3* mutations were detectable in older participants than younger ones. The increasing prevalence of somatic mutations in aged individuals is known [47] and this could speculatively be associated with the gradual worsening of MS, independent of relapses, by age [56], although we emphasize that the findings of this study did not show a link between MS and *STAT3* somatic mutations.

To conclude, our results demonstrate that *STAT3* is a hotspot for somatic mutations in CD8+ cells. The mutations are very commonly detected (in 26% of donors) and suggest a selection advantage of the mutated clones. Although there were no significant differences in the mutation carrier frequencies in MS patients vs. controls, the case on the role of somatic mutations in MS and other common autoimmune diseases is not closed. Our results provide hypotheses for testing in new settings. The discovered high prevalence of somatic mutations in *STAT3* SH2 domain provides opportunities to analyze these mutations directly in autoimmune disease patients’ tissue-infiltrating CD8+ cells. Also, in future studies simultaneous analysis of antigen specificities (e.g. EBV, other viruses, autoantigens) and somatic mutations is warranted to address possible differences between MS and controls at a new level. The larger CD8+ clones are typically targeted against common viruses. EBV-reactive CD8+ T-cells are suspected players in MS, recently identified in MS plaques by using HLA pentamers [57]. An activating somatic mutation in a clone reacting against EBV and CNS antigen by molecular mimicry would be one hypothetical mechanism in MS.

## Supporting information

S1 Fig The sequencing depths of best replicates. (PNG)

S2 Fig The sequencing depths of all replicates. (PNG)

S3 Fig Age vs allelic fraction, mutations from both criteria. (PDF)

S1 Table Participant information, patients. (XLSX)

S2 Table Participant information, controls. (XLSX)

S3 Table Information of the sequenced libraries. (XLSX)

S4 Table All somatic variant calls. (XLSX)

S5 Table Mutation counts by criteria. (XLSX)

S6 Table Logistic regression of phenotype-mutation association. (DOCX)

S7 Table Phenotypic data of patients. (XLSX)

## References

[1] International Multiple Sclerosis Genetics Consortium (IMSGC). Analysis of immune-related loci identifies 48 new susceptibility variants for multiple sclerosis. Nat Genet. 2013;45: 1353–1360. doi: 10.1038/ng.2770 24076602 PMC3832895

[2] International Multiple Sclerosis Genetics Consortium, Patsopoulos NA, Baranzini SE, Santaniello A, Shoostari P, Cotsapas C, et al. Multiple sclerosis genomic map implicates peripheral immune cells and microglia in susceptibility. Science. 2019;365: eaav7188. doi: 10.1126/science.aav7188 31604244 PMC7241648

[3] Bjornevik K, Cortese M, Healy BC, Kuhle J, Mina MJ, Leng Y, et al. Longitudinal analysis reveals high prevalence of Epstein-Barr virus associated with multiple sclerosis. Science. 2022;375: 296–301. doi: 10.1126/science.abj8222 35025605

[4] Xu Y, Hiyoshi A, Smith KA, Piehl F, Olsson T, Fall K, et al. Association of Infectious Mononucleosis in Childhood and Adolescence With Risk for a Subsequent Multiple Sclerosis Diagnosis Among Siblings. JAMA Netw Open. 2021;4: e2124932. doi: 10.1001/jamanetworkopen.2021.24932 34633426 PMC8506233

[5] Young LS, Yap LF, Murray PG. Epstein–Barr virus: more than 50 years old and still providing surprises. Nat Rev Cancer. 2016;16: 789–802. doi: 10.1038/nrc.2016.92 27687982

[6] Goodin DS, Khankhanian P, Gourraud P-A, Vince N. The nature of genetic and environmental susceptibility to multiple sclerosis. Ramagopalan SV, editor. PLoS ONE. 2021;16: e0246157. doi: 10.1371/journal.pone.0246157 33750973 PMC7984655

[7] Gold B. Somatic mutations in cancer: Stochastic versus predictable. Mutation Research/Genetic Toxicology and Environmental Mutagenesis. 2017;814: 37–46. doi: 10.1016/j.mrgentox.2016.12.006 28137366

[8] Abascal F, Harvey LMR, Mitchell E, Lawson ARJ, Lensing SV, Ellis P, et al. Somatic mutation landscapes at single-molecule resolution. Nature. 2021;593: 405–410. doi: 10.1038/s41586-021-03477-4 33911282

[9] Burnet M. Somatic Mutation and Chronic Disease. BMJ. 1965;1: 338–342. doi: 10.1136/bmj.1.5431.338 14237898 PMC2165357

[10] Singh M, Jackson KJL, Wang JJ, Schofield P, Field MA, Koppstein D, et al. Lymphoma Driver Mutations in the Pathogenic Evolution of an Iconic Human Autoantibody. Cell. 2020;180: 878-894.e19. doi: 10.1016/j.cell.2020.01.029 32059783

[11] Goodnow CC. Multistep Pathogenesis of Autoimmune Disease. Cell. 2007;130: 25–35. doi: 10.1016/j.cell.2007.06.033 17632054

[12] Holzelova E, Vonarbourg C, Stolzenberg M-C, Arkwright PD, Selz F, Prieur A-M, et al. Autoimmune Lymphoproliferative Syndrome with Somatic Fas Mutations. N Engl J Med. 2004;351: 1409–1418. doi: 10.1056/NEJMoa040036 15459302

[13] Niemela JE, Lu L, Fleisher TA, Davis J, Caminha I, Natter M, et al. Somatic KRAS mutations associated with a human nonmalignant syndrome of autoimmunity and abnormal leukocyte homeostasis. Blood. 2011;117: 2883–2886. doi: 10.1182/blood-2010-07-295501 21079152 PMC3062298

[14] Beck DB, Ferrada MA, Sikora KA, Ombrello AK, Collins JC, Pei W, et al. Somatic Mutations in UBA1 and Severe Adult-Onset Autoinflammatory Disease. N Engl J Med. 2020;383: 2628–2638. doi: 10.1056/NEJMoa2026834 33108101 PMC7847551

[15] Allegretta M, Nicklas J, Sriram S, Albertini R. T cells responsive to myelin basic protein in patients with multiple sclerosis. Science. 1990;247: 718–721. doi: 10.1126/science.1689076 1689076

[16] Sriram S. Longitudinal study of frequency of HPRT mutant T cells in patients with multiple sclerosis. Neurology. 1994;44: 311–311. doi: 10.1212/wnl.44.2.311 8309581

[17] Valori M, Jansson L, Kiviharju A, Ellonen P, Rajala H, Awad SA, et al. A novel class of somatic mutations in blood detected preferentially in CD8 + cells. Clinical Immunology. 2017;175: 75–81. doi: 10.1016/j.clim.2016.11.018 27932211 PMC5341785

[18] Valori M, Jansson L, Tienari PJ. CD8+ cell somatic mutations in multiple sclerosis patients and controls—Enrichment of mutations in STAT3 and other genes implicated in hematological malignancies. Galli A, editor. PLoS ONE. 2021;16: e0261002. doi: 10.1371/journal.pone.0261002 34874980 PMC8651110

[19] Kuusanmäki H, Dufva O, Parri E, van Adrichem AJ, Rajala H, Majumder MM, et al. Drug sensitivity profiling identifies potential therapies for lymphoproliferative disorders with overactive JAK/STAT3 signaling. Oncotarget. 2017;8: 97516–97527. doi: 10.18632/oncotarget.22178 29228628 PMC5722580

[20] O’Shea JJ, Holland SM, Staudt LM. JAKs and STATs in Immunity, Immunodeficiency, and Cancer. N Engl J Med. 2013;368: 161–170. doi: 10.1056/NEJMra1202117 23301733 PMC7604876

[21] Siegel AM, Heimall J, Freeman AF, Hsu AP, Brittain E, Brenchley JM, et al. A Critical Role for STAT3 Transcription Factor Signaling in the Development and Maintenance of Human T Cell Memory. Immunity. 2011;35: 806–818. doi: 10.1016/j.immuni.2011.09.016 22118528 PMC3228524

[22] Flanagan SE, Haapaniemi E, Russell MA, Caswell R, Allen HL, De Franco E, et al. Activating germline mutations in STAT3 cause early-onset multi-organ autoimmune disease. Nat Genet. 2014;46: 812–814. doi: 10.1038/ng.3040 25038750 PMC4129488

[23] Haapaniemi EM, Kaustio M, Rajala HLM, van Adrichem AJ, Kainulainen L, Glumoff V, et al. Autoimmunity, hypogammaglobulinemia, lymphoproliferation, and mycobacterial disease in patients with activating mutations in STAT3. Blood. 2015;125: 639–648. doi: 10.1182/blood-2014-04-570101 25349174 PMC4304109

[24] Koskela HLM, Eldfors S, Ellonen P, van Adrichem AJ, Kuusanmäki H, Andersson EI, et al. Somatic STAT3 Mutations in Large Granular Lymphocytic Leukemia. N Engl J Med. 2012;366: 1905–1913. doi: 10.1056/NEJMoa1114885 22591296 PMC3693860

[25] Savola P, Brück O, Olson T, Kelkka T, Kauppi MJ, Kovanen PE, et al. Somatic STAT3 mutations in Felty syndrome: an implication for a common pathogenesis with large granular lymphocyte leukemia. Haematologica. 2018;103: 304–312. doi: 10.3324/haematol.2017.175729 29217783 PMC5792275

[26] Jerez A, Clemente MJ, Makishima H, Rajala H, Gómez-Seguí I, Olson T, et al. STAT3 mutations indicate the presence of subclinical T-cell clones in a subset of aplastic anemia and myelodysplastic syndrome patients. Blood. 2013;122: 2453–2459. doi: 10.1182/blood-2013-04-494930 23926297 PMC3790512

[27] Lundgren S, Keränen MAI, Kankainen M, Huuhtanen J, Walldin G, Kerr CM, et al. Somatic mutations in lymphocytes in patients with immune-mediated aplastic anemia. Leukemia. 2021;35: 1365–1379. doi: 10.1038/s41375-021-01231-3 33785863 PMC8102188

[28] Rajala HLM, Olson T, Clemente MJ, Lagstrom S, Ellonen P, Lundan T, et al. The analysis of clonal diversity and therapy responses using STAT3 mutations as a molecular marker in large granular lymphocytic leukemia. Haematologica. 2015;100: 91–99. doi: 10.3324/haematol.2014.113142 25281507 PMC4281318

[29] Jakkula E, Leppä V, Sulonen A-M, Varilo T, Kallio S, Kemppinen A, et al. Genome-wide Association Study in a High-Risk Isolate for Multiple Sclerosis Reveals Associated Variants in STAT3 Gene. The American Journal of Human Genetics. 2010;86: 285–291. doi: 10.1016/j.ajhg.2010.01.017 20159113 PMC2820168

[30] Baranzini SE, Khankhanian P, Patsopoulos NA, Li M, Stankovich J, Cotsapas C, et al. Network-Based Multiple Sclerosis Pathway Analysis with GWAS Data from 15,000 Cases and 30,000 Controls. The American Journal of Human Genetics. 2013;92: 854–865. doi: 10.1016/j.ajhg.2013.04.019 23731539 PMC3958952

[31] Manuel AM, Dai Y, Freeman LA, Jia P, Zhao Z. Dense module searching for gene networks associated with multiple sclerosis. BMC Med Genomics. 2020;13: 48. doi: 10.1186/s12920-020-0674-5 32241259 PMC7118851

[32] Frisullo G, Angelucci F, Caggiula M, Nociti V, Iorio R, Patanella AK, et al. pSTAT1, pSTAT3, and T-bet expression in peripheral blood mononuclear cells from relapsing-remitting multiple sclerosis patients correlates with disease activity. J Neurosci Res. 2006;84: 1027–1036. doi: 10.1002/jnr.20995 16865709

[33] Schneider A, Long SA, Cerosaletti K, Ni CT, Samuels P, Kita M, et al. In Active Relapsing-Remitting Multiple Sclerosis, Effector T Cell Resistance to Adaptive T regs Involves IL-6–Mediated Signaling. Sci Transl Med. 2013;5. doi: 10.1126/scitranslmed.3004970 23363979

[34] Aqel SI, Yang X, Kraus EE, Song J, Farinas MF, Zhao EY, et al. A STAT3 inhibitor ameliorates CNS autoimmunity by restoring Teff:Treg Balance. JCI Insight. 2021 [cited 6 Jun 2022]. doi: 10.1172/jci.insight.142376 33411696 PMC7934926

[35] Hosseini A, Gharibi T, Mohammadzadeh A, Ebrahimi-kalan A, Jadidi-niaragh F, Babaloo Z, et al. Ruxolitinib attenuates experimental autoimmune encephalomyelitis (EAE) development as animal models of multiple sclerosis (MS). Life Sciences. 2021;276: 119395. doi: 10.1016/j.lfs.2021.119395 33781828

[36] Qi Q, Liu Y, Cheng Y, Glanville J, Zhang D, Lee J-Y, et al. Diversity and clonal selection in the human T-cell repertoire. Proc Natl Acad Sci USA. 2014;111: 13139–13144. doi: 10.1073/pnas.1409155111 25157137 PMC4246948

[37] Chen Z, Yuan Y, Chen X, Chen J, Lin S, Li X, et al. Systematic comparison of somatic variant calling performance among different sequencing depth and mutation frequency. Sci Rep. 2020;10: 3501. doi: 10.1038/s41598-020-60559-5 32103116 PMC7044309

[38] Barkhof F. Comparison of MRI criteria at first presentation to predict conversion to clinically definite multiple sclerosis. Brain. 1997;120: 2059–2069. doi: 10.1093/brain/120.11.2059 9397021

[39] Ritari J, Hyvärinen K, Clancy J, FinnGen, Partanen J, Koskela S. Increasing accuracy of HLA imputation by a population-specific reference panel in a FinnGen biobank cohort. NAR Genomics and Bioinformatics. 2020;2: lqaa030. doi: 10.1093/nargab/lqaa030 33575586 PMC7671345

[40] Savola P, Kelkka T, Rajala HL, Kuuliala A, Kuuliala K, Eldfors S, et al. Somatic mutations in clonally expanded cytotoxic T lymphocytes in patients with newly diagnosed rheumatoid arthritis. Nat Commun. 2017;8: 15869. doi: 10.1038/ncomms15869 28635960 PMC5482061

[41] Westman P, Kuismin T, Partanen J, Koskimies S. An HLA-DR typing protocol using group-specific PCR-amplification followed by restriction enzyme digests. Eur J Immunogenet. 1993;20: 103–109. doi: 10.1111/j.1744-313x.1993.tb00099.x 8388248

[42] Li H, Durbin R. Fast and accurate short read alignment with Burrows-Wheeler transform. Bioinformatics. 2009;25: 1754–1760. doi: 10.1093/bioinformatics/btp324 19451168 PMC2705234

[43] Pedersen BS, Quinlan AR. Mosdepth: quick coverage calculation for genomes and exomes. Hancock J, editor. Bioinformatics. 2018;34: 867–868. doi: 10.1093/bioinformatics/btx699 29096012 PMC6030888

[44] Poplin R, Ruano-Rubio V, DePristo MA, Fennell TJ, Carneiro MO, Van der Auwera GA, et al. Scaling accurate genetic variant discovery to tens of thousands of samples. Genomics; 2017 Nov. doi: 10.1101/201178

[45] Sandmann S, de Graaf AO, Karimi M, van der Reijden BA, Hellström-Lindberg E, Jansen JH, et al. Evaluating Variant Calling Tools for Non-Matched Next-Generation Sequencing Data. Sci Rep. 2017;7: 43169. doi: 10.1038/srep43169 28233799 PMC5324109

[46] Schirmer M, Ijaz UZ, D’Amore R, Hall N, Sloan WT, Quince C. Insight into biases and sequencing errors for amplicon sequencing with the Illumina MiSeq platform. Nucleic Acids Research. 2015;43: e37–e37. doi: 10.1093/nar/gku1341 25586220 PMC4381044

[47] Fabre MA, de Almeida JG, Fiorillo E, Mitchell E, Damaskou A, Rak J, et al. The longitudinal dynamics and natural history of clonal haematopoiesis. Nature. 2022 [cited 6 Jun 2022]. doi: 10.1038/s41586-022-04785-z 35650444 PMC9177423

[48] Potapov V, Ong JL. Examining Sources of Error in PCR by Single-Molecule Sequencing. Kalendar R, editor. PLoS ONE. 2017;12: e0169774. doi: 10.1371/journal.pone.0169774 28060945 PMC5218489

[49] Salk JJ, Schmitt MW, Loeb LA. Enhancing the accuracy of next-generation sequencing for detecting rare and subclonal mutations. Nat Rev Genet. 2018;19: 269–285. doi: 10.1038/nrg.2017.117 29576615 PMC6485430

[50] Vu TN, Nguyen H-N, Calza S, Kalari KR, Wang L, Pawitan Y. Cell-level somatic mutation detection from single-cell RNA sequencing. Berger B, editor. Bioinformatics. 2019;35: 4679–4687. doi: 10.1093/bioinformatics/btz288 31028395 PMC6853710

[51] Thomas A, Perzova R, Abbott L, Benz P, Poiesz MJ, Dube S, et al. LGL Leukemia and HTLV. AIDS Research and Human Retroviruses. 2010;26: 33–40. doi: 10.1089/aid.2009.0124 20047475

[52] Kim D, Myllymäki M, Kankainen M, Jarvinen T, Park G, Bruhn R, et al. Somatic STAT3 mutations in CD8+ T cells of healthy blood donors carrying human T-cell leukemia virus type 2. haematol. 2021;107: 550–554. doi: 10.3324/haematol.2021.279140 34706498 PMC8804565

[53] Alagpulinsa DA, Toribio MP, Alhallak I, Shmookler Reis RJ. Advances in understanding the molecular basis of clonal hematopoiesis. Trends in Molecular Medicine. 2022;28: 360–377. doi: 10.1016/j.molmed.2022.03.002 35341686

[54] Niroula A, Sekar A, Murakami MA, Trinder M, Agrawal M, Wong WJ, et al. Distinction of lymphoid and myeloid clonal hematopoiesis. Nat Med. 2021;27: 1921–1927. doi: 10.1038/s41591-021-01521-4 34663986 PMC8621497

[55] Young AL, Challen GA, Birmann BM, Druley TE. Clonal haematopoiesis harbouring AML-associated mutations is ubiquitous in healthy adults. Nat Commun. 2016;7: 12484. doi: 10.1038/ncomms12484 27546487 PMC4996934

[56] Confavreux C, Vukusic S. Natural history of multiple sclerosis: a unifying concept. Brain. 2006;129: 606–616. doi: 10.1093/brain/awl007 16415308

[57] Serafini B, Rosicarelli B, Veroni C, Mazzola GA, Aloisi F. Epstein-Barr Virus-Specific CD8 T Cells Selectively Infiltrate the Brain in Multiple Sclerosis and Interact Locally with Virus-Infected Cells: Clue for a Virus-Driven Immunopathological Mechanism. Longnecker RM, editor. J Virol. 2019;93: e00980–19. doi: 10.1128/JVI.00980-19 31578295 PMC6880158

