# Supplementary figures and images for: High prevalence of low-allele-fraction somatic mutations in STAT3 in peripheral blood CD8+ cells in multiple sclerosis patients and controls
Source: PLoS One. 2022 Nov 28;17(11):e0278245. doi: 10.1371/journal.pone.0278245 (PMC9704626; doi:10.1371/journal.pone.0278245)

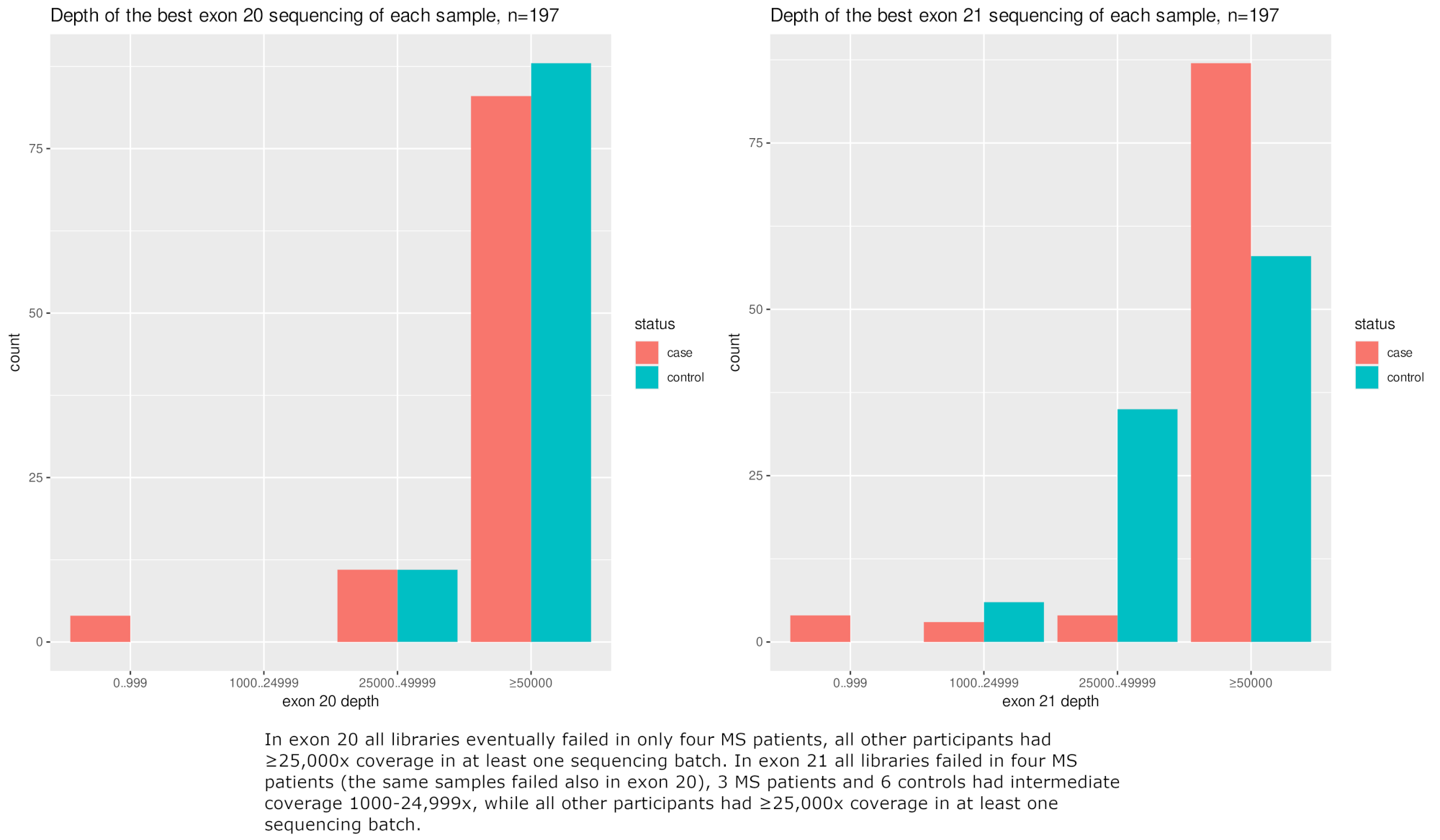

Supplement: S1 Fig — (PNG) [file pone.0278245.s001.png]

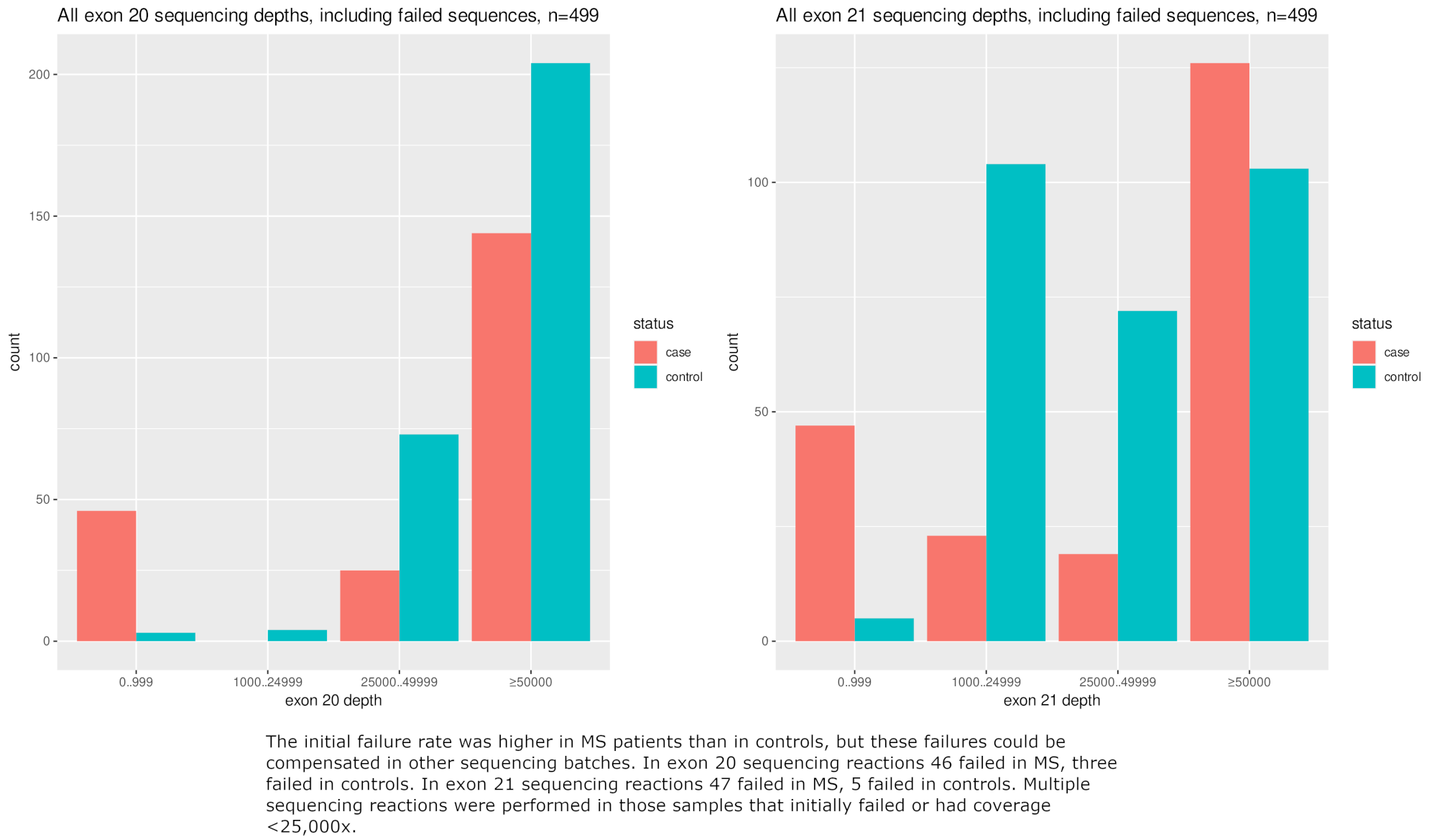

Supplement: S2 Fig — (PNG) [file pone.0278245.s002.png]

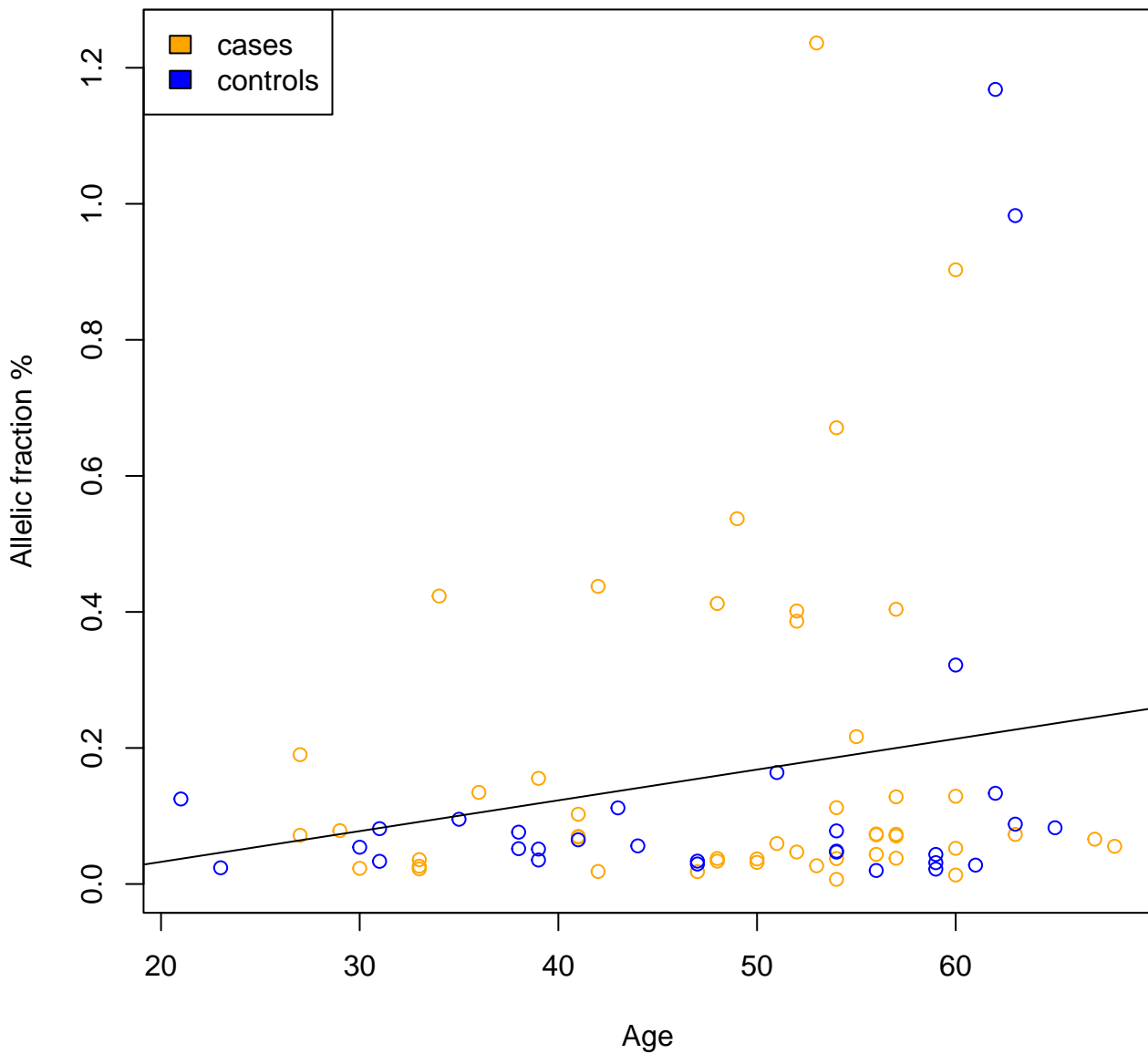

Supplement: S3 Fig — (PDF) [file pone.0278245.s003.pdf]
