# Supplementary material for: High prevalence of low-allele-fraction somatic mutations in STAT3 in peripheral blood CD8+ cells in multiple sclerosis patients and controls
Source: PLoS One. 2022 Nov 28;17(11):e0278245. doi: 10.1371/journal.pone.0278245 (PMC9704626; doi:10.1371/journal.pone.0278245)
Supplement: S6 Table — (DOCX) [file pone.0278245.s009.docx]

Mutation vs no. Multivariate bin log reg.

| **Binary logistic regression, variables in the equation** | | | | | | | |
| --- | --- | --- | --- | --- | --- | --- | --- |
|  | | B | S.E. | Wald | df | Sig. (p) | Exp(B) |
|  | Sex | 1.176 | 0.846 | 1.932 | 1 | 0.165 | 3.240 |
|  | Age | 0.094 | 0.040 | 5.537 | 1 | 0.019 | 1.098 |
|  | BMI | 0.018 | 0.070 | 0.064 | 1 | 0.800 | 1.018 |
|  | Duration from diagnosis | -0.001 | 0.004 | 0.098 | 1 | 0.754 | 0.999 |
|  | DMT | 0.469 | 0.812 | 0.334 | 1 | 0.564 | 1.598 |
|  | Smoking | 1.731 | 0.800 | 4.680 | 1 | 0.031 | 5.648 |
|  | Blood Neutrophiles | -0.588 | 0.250 | 5.522 | 1 | 0.019 | 0.555 |
|  | Constant | -4.550 | 2.651 | 2.947 | 1 | 0.086 | 0.011 |
| a. Variable(s) entered on step: Sex, Age, BMI, Smoking, Duration from diag., Neutr, DMT. BMI = body  mass index, DMT = disease modifying treatment. B = unstandardized regression weight, S.E. = standard error of B, Wald = Wald chi-square, df = degree of freedom from the Wald chi-square test, Exp(B) = exponentiation of B (OR), Sig. = p-value. | | | | | | | |
